# GCNCPR-ACPs: a novel graph convolution network method for ACPs prediction

**DOI:** 10.1186/s12859-022-04771-2

**Published:** 2022-12-23

**Authors:** Xiujin Wu, Wenhua Zeng, Fan Lin

**Affiliations:** 1 School of Informatics, Xiamen University, Xiamen, Fujian China; 2 Computational Health Informatics Program, Boston Children’s Hospital, Boston, MA USA

**Keywords:** Anticancer peptide, Graph convolution network, Graph collapse, Graph representation learning, Classification

## Abstract

**Background:**

Anticancer peptide (ACP) inhibits and kills tumor cells. Research on ACP is of great significance for the development of new drugs, and the prediction of ACPs and non-ACPs is the new hotspot.

**Results:**

We propose a new machine learning-based method named GCNCPR-ACPs (a Graph Convolutional Neural Network Method based on collapse pooling and residual network to predict the ACPs), which automatically and accurately predicts ACPs using residual graph convolution networks, differentiable graph pooling, and features extracted using peptide sequence information extraction. The GCNCPR-ACPs method can effectively capture different levels of node attributes for amino acid node representation learning, GCNCPR-ACPs uses node2vec and one-hot embedding methods to extract initial amino acid features for ACP prediction.

**Conclusions:**

Experimental results of ten-fold cross-validation and independent validation based on different metrics showed that GCNCPR-ACPs significantly outperformed state-of-the-art methods. Specifically, the evaluation indicators of Matthews Correlation Coefficient (MCC) and AUC of our predicator were 69.5% and 90%, respectively, which were 4.3% and 2% higher than those of the other predictors, respectively, in ten-fold cross-validation. And in the independent test, the scores of MCC and SP were 69.6% and 93.9%, respectively, which were 37.6% and 5.5% higher than those of the other predictors, respectively. The overall results showed that the GCNCPR-ACPs method proposed in the current paper can effectively predict ACPs.

## Background

Cancer is a worldwide disease, and the number of people who die of cancer every year is very high [1, 2]. The major strategy for cancer treatment is traditional chemotherapy. Anticancer chemotherapeutic drugs can effectively treat cancer and kill the cancer cells but they can also kill the healthy cells and cause resistance in cancer cells [3]. Therefore, more reasonable and effective therapeutic drugs are urgently needed. Anti-microbial peptides (AMPs) [4] are small molecular peptides produced by organisms and can kill certain cancer cells. Anticancer peptides (ACPs) are typically short peptides containing 10–50 amino acids [5, 6], are a subset of anti-microbial peptides with anticancer activity, and can effectively inhibit tumor growth and kill cancer cells by regulating gene expression [7] and mobilizing the immune system [8]. The ACPs can overcome the shortcomings of traditional cancer treatment methods and can kill cancer cells without harming normal cells, thus, becoming one of the most reliable anticancer therapies. Certain experimental methods have been proposed to determine whether a protein has anticancer activity. However, the main disadvantage of wet-lab experiments is that the process is complex, time-consuming, and costly. In contrast, with the development of machine learning and deep learning methods [9, 10], in silico prediction of the ACPs and non-ACPs has the advantages of being less costly, time-efficient, and highly accurate. A growing number of prediction methods have been proposed that guide the experimental screening of candidate ACPs.

In recent years, the application of machine learning and deep learning models to identify ACPs and non-ACPs has become a research hotspot in the field of bioinformatics and computational biology [11]. More and more ACPs have been discovered and validated experimentally [12]. Most of the previous computational methods use the existing databases, extract the features, and then classify the peptides into ACPs and non-ACPs automatically using the feature training model. For example, Vijayakumar et al*.* proposed a computational method using support-vector machine and protein relatedness measure feature vector [13]. Sequence-based feature extraction methods have been proposed, including amino acid composition, dipeptide composition, and binary pattern, to predict and discover new anticancer peptides [14, 15]. Chen et al*.* reported a sequence-based predictor called iACP, which was developed by optimizing the g-gap dipeptide components to predict the ACPs [16]. Shahid Akbar et al*.* proposed an intelligent model, 'iACP-GAEnsC', based on the evolutionary intelligent genetic algorithm, which uses three different discrete feature representation methods to predict the ACPs [17]. Manavalan et al*.* developed a machine-learning method called MLACP, which used support-vector machine and Random Forest (RF)-based tool to predict the ACPs using the amino acid sequence features [18]. Wei et al*.* proposed the ACP-FL method, which extracted and learned a set of informative features of the protein from a pool of support-vector machine-based models to identify the ACPs [19]. Another method called QSPred-FL [20] has also been proposed to automatically learn the most discriminative features from the existing feature descriptors in a supervised way to classify the ACPs and non-ACPs. Wei et al*.* further designed a bioinformatics tool for the ACP prediction called PEPred-Suite [21]. The PEPred-Suite extracted diverse sequence-based features, which could reliably predict different ACPs using RF models.

With the development of more and more ACP-predicting methods, their prediction accuracy has increased. The improved predictive ability of various computational methods will rapidly push forward their applications in cancer therapy. Graph neural network (GNN) is an advanced deep learning model that has been applied for various bioinformatics tasks [22–24], such as link prediction [11], node classification, and community detection. More and more GNN methods [25] are proposed, and their application to the ACP data can be considered. Most of the ACP data consisted of sequence-based features, and it was then integrated with the other features as the classifier inputs to build the predictive model. However, most of the traditional classification models for ACP prediction mainly regard ACP data as ordered sequence data, ignoring the ACP structure and the relationship between amino acids. If the ACP data was regarded as a kind of structured data, likely graph data, the amino acids would be regarded as nodes and the relationship between amino acids as edges. Then, we could use graph-based methods to deal with ACP data. Moreover, the physicochemical information will be integrated to describe the node attributes in the graph, making the ACPs a topological map composed of amino acid sequences.

In the current study, a graph convolution network (GCN) method based on graph collapse pooling and residual network for predicting ACPs (GCNCPR-ACPs) was used to deal with the graph-based data of ACPs. The graph convolutional neural network is used to extract the graph structure features of amino acids and calculate the graph collapse pooling operator. The graph collapse pooling module is used to aggregate multiple nodes into a large node. After several layers of collapse, the peptide chain composed of amino acids graph is finally collapsed into a large node, and the feature of the collapsed large node is the feature of the whole ACP line. The residual network is used to solve the problem of gradient disappearance caused by the deepening of layers. The main contributions of the current study can be summarized as follows:

1. We proposed a novel GCN-based framework named GCNCPR-ACPs for ACP prediction. To the best of our knowledge, this is the first attempt to adapt graph collapse pooling for ACP prediction.
2. To effectively capture different levels of node attributes for amino acid node representation learning, this is the first attempt to use 4 kinds of node attributes for ACP prediction, containing 2 physicochemical properties of amino acids and 2 others extracted by one-hot embedding and node2vec methods separately.
3. The GCNCPR-ACPs method predicts the ACPs based on multiple properties of the nodes, the diverse characteristics of the ACP graph data, and the novel GCNCPR model.
4. Experiments have been conducted extensively to evaluate the performance of the graph convolutional neural network method based on collapse pooling and residual network.

## Results and discussion

In this section, we introduced the evaluation metric used in our experiments; then we presented the evaluation of the performance of the proposed GCNCPR-ACPs model using these evaluation indicators, and discussed the results. Finally, we introduced the experimental setting parameter to verify the effectiveness of the model.

### Evaluation metric

For performance evaluation, several machine learning metrics are widely used in prediction methods. They were used to verify the effectiveness of our model, including sensitivity (SE), specificity (SP), accuracy (ACC), Matthew’s correlation coefficient (MCC), and area under the curve (AUC). The formulas of the five metrics used are as follows:

1 $$\left\{ {\begin{array}{*{20}l} {Sensitive = recall = \frac{TP}{{TN + FN}} = \frac{TP}{P} \times 100\% } \hfill \\ {Specificity = \frac{TN}{{TN + FP}} = \frac{TN}{N} \times 100\% } \hfill \\ {Precision = \frac{TP}{{TP + FP}} \times 100\% } \hfill \\ {Accuracy = \frac{TP + TN}{{TP + TN + FP + FN}} = \frac{TP + TN}{{P + N}} \times 100\% } \hfill \\ {MCC = \frac{TP \times TN - FP \times FN}{{\sqrt {(TP + FN)(TP + FP)(TN + FN)(TN + FP)} }} \times 100\% } \hfill \\ {F1 = \frac{2 \times recall \times precision}{{recall + precision}}} \hfill \\ \end{array} } \right.$$

True positive (TP) indicates the number of true ACP samples that are predicted correctly. False positive (FP) indicates the number of ACP samples with false prediction, that is, the non-ACP samples that are classified as ACP samples by the classifier. True negative (TN) indicates the number of non-ACP samples that are predicted correctly. False negative (FN) indicates the number of non-ACP samples with false prediction. AUC measures the overall performance of the predictor. The higher the AUC, the better is the performance of the model.

### Results of ten-fold cross-validation

To validate the predictive performance of the proposed GCNCPR-ACPs, we compared its performance with that of several existing predictors, including iACP [16], ACPred-FL [19], PEPred-Suite [21], ACPred-Fuse [26], AntiCP_ACC [13], AntiCP_DC [13], and Hajisharifi’s [27]. The cross-validation results are presented in Table 1. It was observed that GCNCPR-ACPs outperformed, since the scores of all of its evaluation indicators were the highest, especially, the Matthews Correlation Coefficient (MCC) and specificity (SP), which were 88% and 100%, respectively.

**Table 1:** Cross-validation results of the GCNCPR-ACPs and other methods

Methods	SE	SP	ACC	MCC	AUC
iACP	57.2	84.0	70.6	42.8	80.9
ACPred-FL	71.6	84.4	78.0	56.5	84.6
PEPred-Suite	72.8	88.0	80.4	61.5	86.0
ACPred-Fuse	77.2	87.6	82.4	65.2	88.2
AntiCP_ACC	66.8	78.4	72.6	45.5	82.4
AntiCP_DC	71.6	77.6	74.6	49.3	82.5
Hajisharifi’s	67.2	83.6	75.4	51.5	83.1
GCNCPR-ACPs	**81.5**	**88.1**	**84.6**	**69.5**	**90.0**

The highest scores are marked in bold and the second-highest are underlined

## Results of independent test

To validate the robustness of the proposed GCNCPR-ACPs, we compared its performance with that of several existing predictors. In the independent test, the scores of MCC and SP of our model were 69.6% and 93.9%, respectively, and were 37.6% and 5.5% higher than that of the other predictors, respectively. The independent test results are presented in Table 2.

**Table 2:** Independent test results of the proposed predictor and the existing predictors

Methods	SE	SP	ACC	MCC	AUC
iACP	54.9	88.8	87.7	22.6	76.1
ACPred-FL	69.5	85.8	85.3	25.9	85.1
PEPred-Suite	68.3	90.6	**89.9**	32.0	86.1
ACPred-Fuse	72	89.5	89	32.0	**86.8**
AntiCP_ACC	68.3	88.5	87.9	28.8	85.3
AntiCP_DC	68.3	82.6	82.2	22.3	83.0
Hajisharifi’s	69.5	88.4	87.9	29.2	85.5
GCNCPR-ACPs	**74.4**	**93.9**	84.1	**69.6**	84.1

The highest scores are marked in bold and the second-highest are underlined

### Parameter analysis

Several important parameters influence the performance of our model, such as the learning rate, the number of layers of the GCN, and the assign ratio. In the current section, we present the results of the sensitivity analysis of these parameters. In our model, the training epoch was set to 1000. The hidden dimension and the output dimension were 64.

**Fig. 1:**
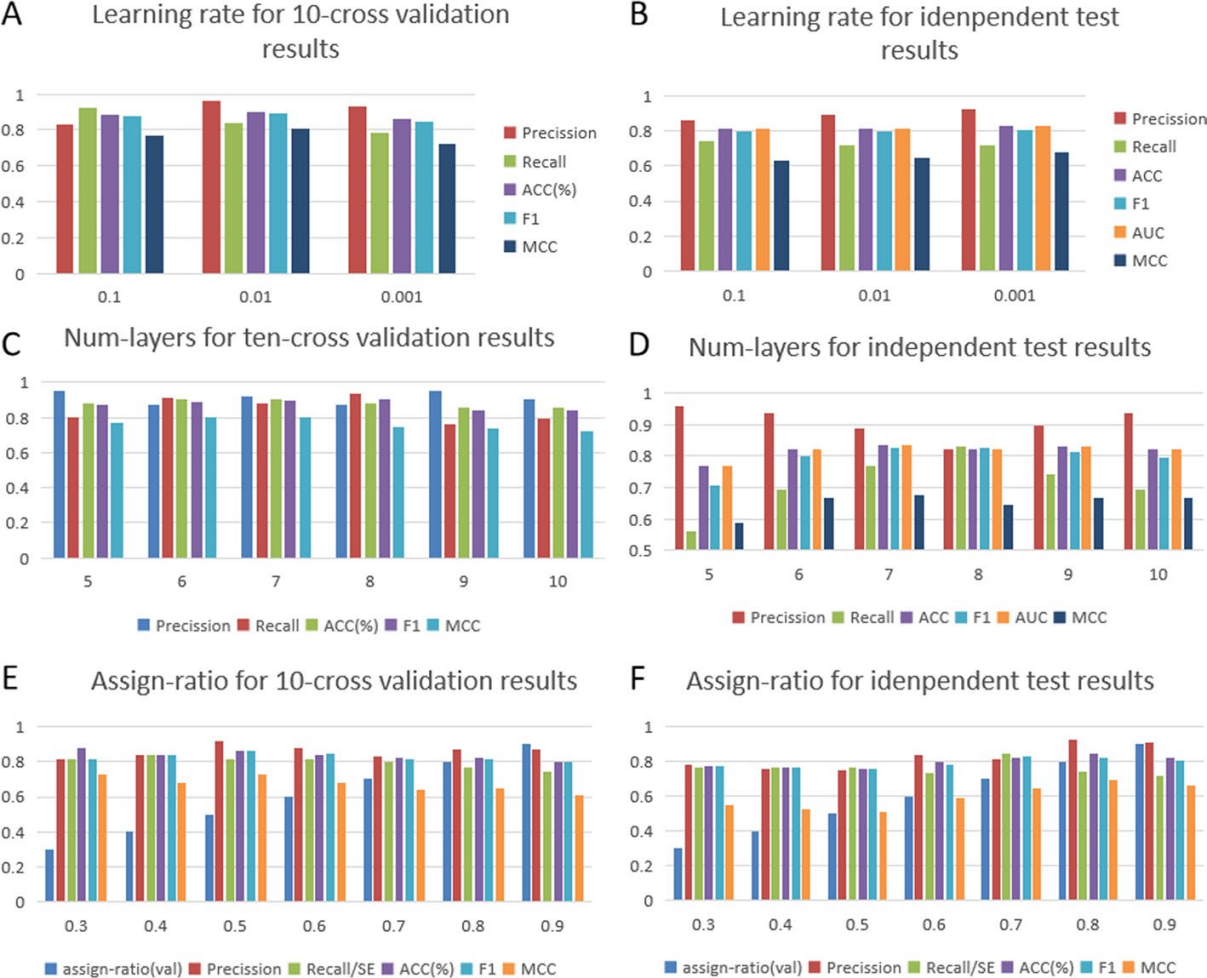
The comparison of the performance of our proposed GCNCPR-ACPs with other state-of-the-art predictors. **A** The effects of the learning rate on the performance of the tenfold cross-validation results of the proposed GCNCPR-ACPs and the existing prediction models. **B** The effects of the learning rate on the performance of the independent test results of the proposed GCNCPR-ACPs and the existing prediction models. **C** The effects of the number of layers on the performance of the tenfold cross-validation results of the proposed GCNCPR-ACPs and the existing prediction models. **D** The effects of the number of layers on the performance of the independent test results of the proposed GCNCPR-ACPs and the existing prediction models. **E** The effects of the assign ratio on the performance of the tenfold cross-validation results of the proposed GCNCPR-ACPs and the existing prediction models. **F** The effects of the assign ratio on the performance of the independent test results of the proposed GCNCPR-ACPs and existing prediction models

We then evaluated our model by choosing the learning rate from 0.1, 0.01, and 0.001. Figure 1A, B show that as the learning rate varies, the performance gradually increases initially and then decreases, where a learning rate of 0.01 gives the best performance. As shown in Fig. 1C, D, we observed that our model was slightly influenced by the number of layers. After increasing the number of layers from 5 to 10 with a step value of 1, we observed that our model was relatively robust since ACC and F1 were quite stable. The assign ratio was chosen from 0.3, 0.4, 0.5, 0.6, 0.7, 0.8, and 0.9. As shown in Fig. 1E, F, when the assign ratio was larger, the performance of independent test results was better.

### Ablation experiments

We compared our experiments with ablation experiments. The training dataset was divided using a ratio of 9:1, that is, into 450 training samples and 50 verification samples for the ablation experiment. The ablation experiment results are presented in Table 3. We observed that our experiment was mainly composed of three modules: n-layer stacking graph convolution neural network G(X), graph collapse pooling module D(X), and residual network R(X). We observed that the results of our model were the best, the results of G(X) + D(X) were second-to-best, and the results of G(X) were the worst.

**Table 3:** Results of the ablation experiments

Model	Precision	Recall	ACC	F1	MCC
G(X)	0.76	0.75	0.76	0.76	0.45
G(X) + R(X)	0.83	0.83	0.84	0.83	0.67
G(X) + D(X)	0.83	0.74	0.84	0.78	0.62
G(X) + D(X) + R(X)	0.84	0.86	0.88	0.85	0.70

### Training ratio comparison experiments

For comparison, the setting of training data set is the same as that of Wei's articles, and they all focus on anticancer peptides prediction [28, 29]. The training data was balanced, i.e., there were 250 positive samples and 250 negative samples. We also tried different ratios of positives-to-negatives (e.g., 1:1, 1:5, and 1:10) to further test the performance of the proposed model. And the training ratio comparison experiments results are presented in Table 4. We observed that the training ratio of 1:1 worked best, the training ratio of 1:5 worked second-to-best, and the training ratio of 1:10 worked the worst. As the proportion of positive and negative training samples changes from 1:1 to 1:10, the experimental results show that with the ratio between positive and negative samples decreases, the recall increases, but all of the other values decrease.

**Table 4:** Results of the training ratio comparison experiments

Ratio	Precision	Recall	ACC	F1	MCC
1:1	0.84	0.86	0.88	0.85	0.70
1:5	0.61	0.96	0.70	0.75	0.49
1:10	0.47	1.0	0.54	0.63	0.38

## Conclusion

Here, we proposed a new prediction model called GCNCPR-ACPs. It is a powerful bioinformatics tool to predict anticancer peptides using GCN and graph collapse pooling and residual network model. The advantage of GCNCPR-ACPs is that it can effectively construct the anticancer peptide map. It can extract useful features from graph data, including node attributes, line attributes, icon labels, node labels, and adjacency matrix. GCNCPR-ACPs model is novel and it mainly includes the following modules: graph differentiable pool module D(X), stacked graph convolution neural network module G(X), and residual network module R(X). The experimental results of ten-fold cross-validation and independent test show that the proposed predictor can more effectively classify the ACPs and non-ACPs. The effective predicting ability of the model will accelerate its application in cancer treatment.

## Methods

**Fig. 2:**
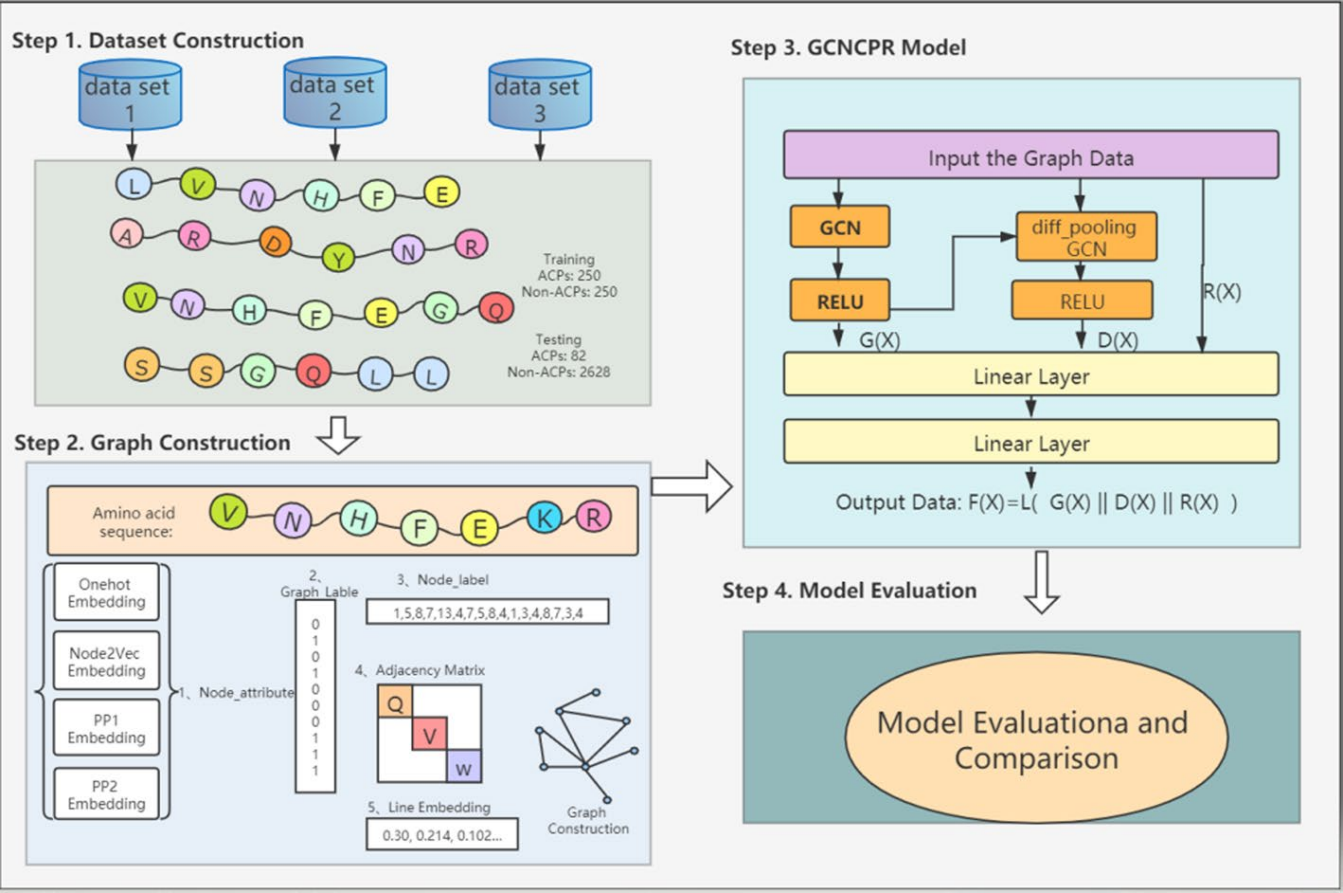
The overview of the GCNCPR-ACPs predictor. Step 1, data construction: the ACP datasets are prepared to obtain the training and test datasets. Step 2, graph construction: the ACPs chains are used to construct the graphs using the amino acids as nodes. Step 3, GCNCPR model: the graph data is used as input for the GCNCPR model and to classify the ACP chain. Step 4, model evaluation: the classification results of our model are evaluated and compared with those of the other models

In the current section, we will introduce the overall framework of our model. The steps of the model are shown in Fig. 2. Step 1: the amino acid sequences of ACPs are collected from three protein datasets to form our training and test datasets. Step 2: the ACP chains will be used to construct the graphs with the amino acids as nodes. The graph data properties contain the graph labels, node labels, adjacency matrix, node attributes, and line embedding data. Step 3: the GCNCPR model will be introduced in detail. Step 4: The training and test results of our model will be discussed and compared with those of other models.

### Materials

The peptides with anticancer activity are called anticancer peptides (ACPs), and they are regarded as positive samples. On the contrary, the peptide samples without anticancer activity are called non-anticancer peptides (non-ACPs) and are considered negative peptides. In the current chapter, the training and independent test datasets of ACPs are introduced.

In the current study, we used the datasets used by Wei et al*.* [19]. There were 332 ACP samples and 2878 non-ACP samples in the dataset. In the report by Wei et al*.* [19], the training datasets contained 250 ACPs and 250 non-ACPs. The rest of the dataset included the remaining 82 ACP samples (positive samples) and 2628 non-ACP samples (negative samples).

### Graph construction

The ACPs are composed of amino acid sequences. According to graph neural network theory, the amino acids are regarded as vertices (V) and the links between amino acids as edges (E). The ACP protein chain, which is composed of amino acid sequences, is used to construct the ACP graph neural network (G). The ACP graph network (G) possesses the graph data properties, such as node attributes, line attributes, graph labels, node labels, and the adjacency matrix A. ${\text{A}}_{{{\text{n}} \times {\text{n}}}} \in [0,{ }1]$. The number “1” denotes that the two amino acids are connected and “0” denotes that there is no edge between them. The graph labels are the labels of the ACP chains that are composed of several amino acids. The graph label value of “1” denotes non-ACPs (negative samples), and “0” denotes ACPs (positive samples). The line attribute, as proposed by Wei et al*.* [19], represents the characteristic of one ACP chain. The amino acid nodes in the graph are represented by 20 English letters. The amino acid nodes are divided into 20 categories, and each amino acid node has a category identifier from 1 to 20, called node labels. The node attributes are the features of amino acid nodes in the graph, obtained using four embedding methods—one-hot embedding method, node2vec embedding method, and two kinds of physicochemical property-embedding methods.

### One-hot embedding

One-hot embedding is an effective encoding method expressed using binary vectors. Only one bit is valid at any time, and other positions are set to 0. The primary structural information of ACP protein is mainly composed of 20 common amino acids. Each of these 20 amino acids is represented by a single English letter (A, C, D, E, F, G, H, I, K, L, M, N, P, Q, R, S, T, V, W, and Y). Therefore, each amino acid node in the graph is represented as a 20-dimensional feature vector by one-hot embedding.

### Node2vec embedding

**Fig. 3:**
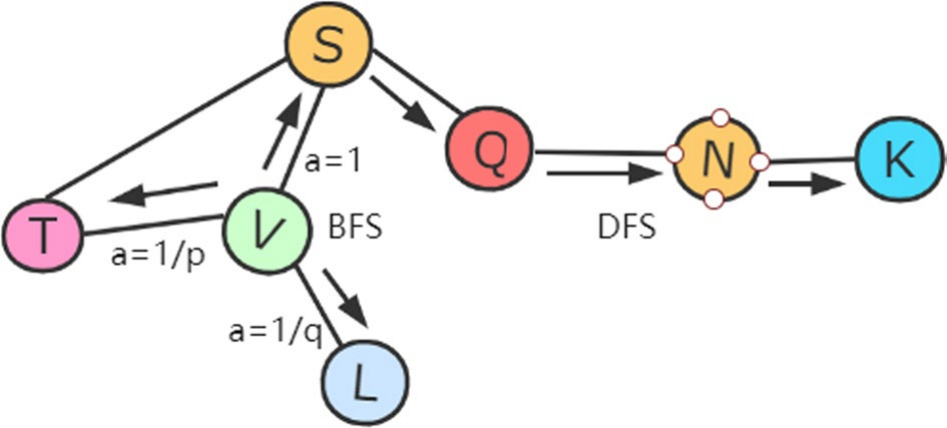
Node2vec embedding method. Shows the BFS and DFS search strategies starting from node V. In the current study, we used the DFS sampling method to sample the amino acid nodes

Node2vec embedding considers the distance between two nodes. We used the node2vec embedding method to encode amino acid nodes. Node2vec uses random walk to get the nearest neighbor information of the vertices. The Node2vec embedding method mainly uses random walk to sample the node sequence. When it comes to random walk sampling, there are two main kinds of graph walking—depth-first sampling (DFS) and breadth-first sampling (BFS), shown in Fig. 3.

### Physicochemical property embedding

Although the above embedding methods can consider the characteristics of the nodes in the graph and evaluate the distance between two nodes, the amino acid nodes have their own physical and chemical sense. To capture the physical and chemical sense of the amino acid node, Dou et al. [30] divided the 20 amino acids into 10 groups according to the physicochemical properties of these amino acids. The amino acids often have more than one property. As shown in Table 5, the ten properties are binary coded to form a 10-dimensional embedding feature vector.

**Table 5:** The categorization of the standard amino acid nodes based on ten physicochemical properties

Id	Physicochemical properties	Amino acids
1	Aromatic	F, Y, W, H
2	Negative	D, E
3	Positive	K, H, R
4	Polar	N, Q, S, D, E, C, T, K, R, H, Y, W
5	Hydrophobic	A, G, C, T, I, V, L, K, H, F, Y, W, M
6	Aliphatic	I, V, L
7	Tiny	A, S, G, C
8	Charged	K, H, R, D, E
9	Small	P, N, D, T, C, A, G, S, V
10	Proline	P

The second embedding feature method based on the physicochemical properties of amino acids is shown below. According to the Composition, Transition, and Distribution (CTD) [31] of the amino acid attributes, the standard amino acids can be categorized using seven physicochemical properties as shown in Table 6. Each physicochemical property has three groups. Therefore, a total of 21 embedding features are used to characterize each amino acid node.

**Table 6:** The categorization of the standard amino acid nodes based on the seven physicochemical properties

Physicochemical properties	Group 1	Group 2	Group 3
Hydrophobicity	A, C, F, G, H, I, L, M, N, P, Q, S, T, V, W, Y	D, E	K, R
Normalized	C, F, I, L, M, V, W	A, G, H, P, S, T, Y	D, E, K, N, Q, R
Polarity	A, C, D, G, P, S, T	E, I, L, N, Q, V	F, H, K, M, R, W, Y
Polarizibility	C, F, I, L, M, V, W, Y	A, G, P, S, T	D, E, H, K, N, Q, R
Charge	A, D, G, S, T	C, E, I, L, N, P, Q, V	F, H, K, M, R, W, Y
Secondary structures	D, G, N, P, S	A, E, H, K, L, M, Q, R	C, F, I, T, V, W, Y
Solvent accessibility	A, C, F, G, I, L, V, W	H, M, P, S, T, Y	D, E, K, N, R, Q

### GCNCPR model

**Fig. 4:**
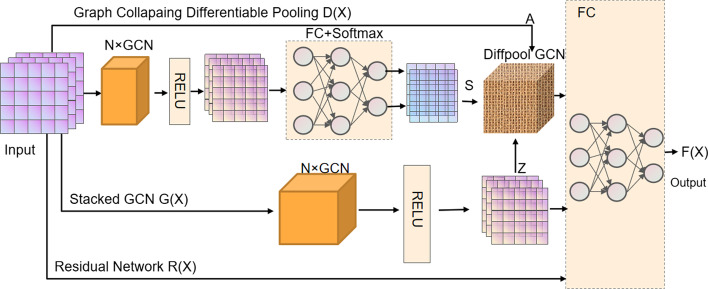
The GCNCPR module. The GCNCPR model mainly includes the following modules—the graph collapse differentiable pooling module D(X), the stacked graph convolution neural network module G(X), and the residual network module R(X)

In the current section, we introduced a flexible model using an n-layered GCN for supervised learning of a graph (Fig. 4). Firstly, the graph data is input into the GCNCPR module. The stacked graph convolution neural network module G(X) is used to extract the features of ACPs, the graph collapse differentiable pooling module D(X) is used to extract the ACP chain features, and the residual network R(X) is used to prevent the gradient disappearing problem. We combined these ACP features and input them into the FC (full connection) module for dimension reduction to get the final output data. Then, the ACP chains were classified into ACPs and non-ACPs. We then concatenated the learned representations as the ACP chain features as below:

2 $${\text{F(X)}} = {\text{L(G(X)}}\left\| {\text{D(X)}} \right\|{\text{R(X))}}$$

where || denotes the operation of vector concatenation, such as mean or addition, and L(.) represents the function that passes through two linear fully connected layers.

### Stacked graph convolution network G(X)

The stacked graph convolution network is a multi-layered stack of GCN, which is used to learn the representation of amino acid nodes in the ACP graph. The theoretical formula of a single layer of GCN is as follows:

3 $$H^{{\left( {l + 1} \right)}} = \sigma \left( {\tilde{D}^{{ - \frac{1}{2}}} \tilde{A}\tilde{D}^{{ - \frac{1}{2}}} H^{\left( l \right)} W^{\left( l \right)} } \right)$$

where $H^{(l)} \in R^{n \times d}$, n is the number of nodes in the graph, and each node is represented by a d-dimensional feature vector called node attribute. The input feature of layer $l$ is $H^{\left( l \right)}$. *A* is an adjacency matrix. $\tilde{A}$ is the adjacency matrix with self-connections,$\tilde{A} = A + I_{N}$.$\tilde{D}$ is the degree matrix, $\tilde{D}_{ij} = \sum\nolimits_{j} {\tilde{A}_{ij} }$.$W^{\left( l \right)}$ is the trainable parameter, and $\sigma$ is the corresponding activation function, such as ${\text{ReLU}}( \cdot )$ or $max(0, \cdot )$. $H^{(l + 1)}$ is the output feature data of the (*l* + 1)th layer graph. Equation (2) is the final form of GCN.

### Graph collapsing differentiable pooling module D(X)

Differential pooling is an algorithm that combines the graph collapse process with GCN for graph representation learning [32]. Firstly, the graph data with amino acid node features ${\text{H}}^{(l)}$ and adjacency matrix ${\text{A}}^{(l)}$ of the nodes in layer ${\text{l}}$ were input into the differential pooling GCN module. According to the GCN formula above, after three layers of GCN, we get the node feature expression ${\text{Z}}^{(l)}$. The softmax is used to make a full connection, the connection structure of the lower layer supernodes is obtained, and the matrix allocator ${\text{S}}^{(l)}$ is learned, whose value represents the probability that the nodes are assigned to any cluster. The matrix allocator ${\text{S}}$ is also called the graph collapse operator. The closer the probability value, the more likely are the nodes to be assigned to the same cluster.

4 $$Z^{\left( l \right)} = H^{{\left( {l + 1} \right)}} = G_{l,embed} \left( {A^{\left( l \right)} ,H^{\left( l \right)} } \right)$$

5 $$S^{\left( l \right)} = softmax\left( {G_{l,pool} \left( {A^{\left( l \right)} ,H^{\left( l \right)} } \right) } \right)$$

$G_{l,embed}$ and $G_{l,pool}$ are two independent ${\text{GCN}}$ layers. Their inputs are the same, which are the amino acid node features ${\text{H}}^{(l)}$ and adjacency matrix ${\text{A}}^{(l)}$ of the nodes. However, their parameters and learning purposes are different. For the cluster allocation matrix ${\text{S}}$ in the last layer, we need to directly fix it into a matrix, which is filled by “1”, because we need to collapse the graph into a super large node, to obtain the global representation of the graph.

6 $$Z^{{\left( {l + 1} \right)}} = S^{{\left( l \right)^{T} }} Z^{\left( l \right)}$$

7 $$A^{{\left( {l + 1} \right)}} = S^{{\left( l \right)^{T} }} A^{\left( l \right)} S^{\left( l \right)}$$

where ${\text{A}}^{{\left( {\text{l}} \right)}} \in {\text{R}}^{{{\text{n}}^{{\left( {\text{l}} \right)}} \times {\text{n}}^{{\left( {\text{l}} \right)}} }} ,{\text{ S}}^{{\left( {\text{l}} \right)}} \in {\text{R}}^{{{\text{n}}^{{\left( {\text{l}} \right)}} \times {\text{n}}^{{\left( {{\text{l}} + 1} \right)}} }} ,{\text{ n}}^{{\left( {\text{l}} \right)}}$ represents the number of nodes in layer ${\text{l}}$, ${\text{A}}^{{\left( {{\text{l}} + 1} \right)}}$ and ${\text{Z}}^{{\left( {{\text{l}} + 1} \right)}}$ denotes the number of nodes (clusters) in layer $\left( {{\text{l}} + 1} \right)$.

### Residual network module R(X)

It is known that increasing the depth of the network improves the performance of the network. The performance of a shallow neural network is often poor than that of a deep neural network, but if we simply increase the depth, it will lead to gradient dispersion or gradient explosion. Moreover, with the increase in network layers, the accuracy of the training set does not increase further or even decreases, resulting in degradation. To solve this problem, we use the residual network into our model.

### Loss function

Cross entropy loss function was used to optimize the model training loss. In the case of two classifications, the final prediction results of the model are only two cases, either 0 or 1. For each category, the probability of our prediction is p and 1 − p. The specific formula used was as follows:

8 $$Loss = - \frac{1}{N}\sum\limits_{i} { - [y_{i} log(p_{i} ) + (1 - y_{i} ) \cdot log(1 - p_{i} )]}$$

where, $y_{i}$ represents the real label of the *i*th sample. And $p_{i}$ represents the probability of the *i*th sample which is predicted to be a positive class. N represents the number of all of the ACP samples.

## Data Availability

ACP500 and ACP164 datasets are available at https://github.com/hengenggg/gcncpr.

## Abbreviations

ACP: Anticancer peptide
GCN: Graph convolution network
GCNCPR-ACPs: The graph convolutional neural network method based on collapse pooling and residual network (GCNCPR) for ACPs prediction
CTD: Composition, transition, and distribution
